# Inaugural Address, Delivered at a Meeting of the Medico-Botanical Society of London, Held February 16th, 1831; Philip Henry Earl Stanhope, President, in the Chair

**Published:** 1833-02

**Authors:** Gilbert T. Burnett

**Affiliations:** Professor of Botany to the Society, and Lecturer in the School of Physic, St. George's Hospital, &c. &c. &c.


THE LONDON

### Medical and Physical Journal.

408, VOL. LXIX.] FEBRUARY 1833. [80, *New Series.*

"For many fortunate discoveries in medicine, and for the detection of numerous errors, the world is indebted to the rapid circulation of Monthly Journals; and there never existed any work to which the Faculty in Europe and America, were under deeper obligations than to the 'Medical and Physical Journal of London,' now forming a long but an invaluable series."—RUSH.

### ORIGINAL PAPERS.

TRANSACTIONS OF THE MEDICO-BOTANICAL SOCIETY OF LONDON.

*Printed under the Direction of the President and Council.*

### INAUGURAL ADDRESS.

*Inaugural Address, delivered at a Meeting of the Medico-Botanical Society of London, held February* 16*th*, 1831; PHILIP HENRY EARL STANHOPE, *President, in the chair.* By GILBERT T. BURNETT, Professor of Botany to the Society, and Lecturer in the School of Physic, St. George's Hospital, &c. &c. &c.

MY LORD PRESIDENT, AND GENTLEMEN:

As circumstances vary the relations in which men stand, the duties are varied they are destined to perform. Each step advanced, each height attained, as it expands their influence, in an equal degree extends their care. Posts of honour are not posts of ease, but are regarded in their truest light when they are viewed as stations of responsibility; and before attempting to fulfil the duties of such stations, all, who sincerely desire to discharge them with credit and profit to themselves and others, should endeavour clearly to understand their nature, and to become fully impressed with their importance. Since, therefore, being called to this honourable chair, I have laboured (not wholly, it is hoped, with fruitless effort,) to inform myself as to what must be required of the Professor of Botany to the Medico-Botanical Society; an office of no mean rank, in a Society of no mean importance: a Society, the bare mention of whose objects must immediately challenge our attention and regard, directed, as its exertions are, to the philosophic investigation of the distinctive characters and medicinal properties of plants; a knowledge by which, when fully gained, the power of physic will be much increased, and pain disarmed of half its terrors, by which *(Deo volente!)* the life of man may be much prolonged, and human misery may much be lessened.

As the avowed objects for which this Society was instituted, and the laws by which the Council govern, alike preclude all dissertations on purely botanical researches, topics upon which a botanic mind luxuriates, points upon which it loves to expatiate; of course, all comments on the laws of life affecting the development of the vegetable structure; all remarks on the wonderful metamorphoses, both regular and irregular, transitional and vicarious, which plants continually pass through; all disquisitions on the vital functions of these simplest organic beings; the assimilation of their food; the production of their peculiar principles; the reproduction of their species; their irritability; their seeming instincts; with many, many other curious and interesting physiological themes, though pregnant with information and replete with wonder, are all alike forbidden here. Botany being, to the Medico-Botanical Society, but one among several means by which it proposes to advance an ulterior object; hence, I must confess that my favorite study must not be allowed undue preponderance, and I submit to the decision, that, although a *means*, it is not *here* an *end;* it is one way to the science here held in view, (and a delightful flowery path it is,) but that it must not be mistaken for the goal itself.

Therefore, as these favorite topics would be here irrelevant, to a not less important, though less entrancing section must our attention at this present be directed, to one subdivision of one section of this extensive science, viz. Economic Botany; whence I shall be led to discuss the uses and properties of vegetables, more especially of medicinal plants, and medicinal preparations. Hence, in order not to wander from my province, and hoping to be set right by the Council if I greatly err, is it that I have resolved, in this my inaugural address, to trace, as it were in outline, the progress of medico-botanical science; for thus will be most clearly seen, not only the benefits it has bestowed upon mankind,—for thus will be most certainly foreshown, not only the blessings it has still in store, but also, from this conspectus of what has been already done, we shall be directed to what there remains for us to do: directed to that course which may most advantageously be pursued, and encouraged in those researches by which, in conjunction with his colleagues, the professor of botany may hope most efficiently to serve the ends, most rapidly to advance the objects, of your Society: for he feels, acutely feels, that, as an office is honourable, so, in an equal degree, does responsibility attach to him that holds it.

Gentlemen: As long as the human race have been subject to disease, as long as pain has been an evil, so long must means of alleviation have been sought, and so long must medicines have been prescribed and used. Rude, indeed, were the early essays of our art, and long must they have continued rude: the morning twilight of physic has been for ages dawning into perfect day; comparatively, it is not long since men, ignorant alike of the indications to be observed, and of the instruments by which those indications might be fulfilled, prescribed scarlet clothes for fever, because they both were red, and saffron for jaundice, on account of its yellow hue.

Much lately has been done in the investigation of diseases, their causes, their symptoms, and their effects; pathological anatomy has revealed many of the changes which various structures undergo, some of which morbid conditions impair the energies, and others are incompatible with the duration of life. But what avails it that the physician can trace by symptoms the successive stages of disorganization, as they proceed in structures concealed from view? what avails it that the surgeon can proclaim the appearance of such morbid alterations long before dissection unfolds them to the light? what avails it that both can foretell the impairment or destruction of vital parts, without they can at the same time learn to check the ravages of disease, and either to alleviate the sufferings of the patient or to afford him a perfect cure? Without such an application of this art, the means of obtaining it would to many be repulsive, and the science itself not a blessing, but a bane; as the foreknowledge of ills that could not be relieved would but aggravate the misery man is called on to endure. But such is not the opprobrium of our useful, and hence noble, arts; for the theory of physic is founded on experience, and the benefits of its practice who can venture to deny! As sciences, medicine and surgery find few their equals; and as arts they are excelled by none.

Much, I repeat, has been lately done to advance our knowledge of disease, and something, though much less, to perfect our instruments of cure. These, however, are sister-sciences, or rather their connexion is of a still closer kind: they are twins, which, as they are naturally, so for ever they should remain inseparably conjoined; and for the Medico-Botanical Society, whose attention is especially directed to this point, has the grateful task been left of perfecting this union: on it devolves the duty of steadying the arm of science, and placing surer weapons in her hands; i. e. of providing for physic new and more certain remedial means.

A circumstance which still shrouds medicine in mystery, must have been formerly much more perplexing than we find it now. Even, however, in the present day, it frequently involves the principles of our practice in obscurity; and hence some persons, ignorant of how many cases there are in which it approaches demonstration, have not scrupled to call physic a conjectural science; to define its object to be the calculation of chances, and its decision the balance of probabilities. I, of course, allude to the acknowledged difficulty of determining how far a cure should be attributed to the renovating powers of life, and how far to the remedial agents which art employs: for some diseases, and especially in some constitutions, will disappear not only without, but even in spite of, the physician; whilst others, in other persons, or even in the same person at other times, not the most consummate skill can cure. Of this, the records of legitimate practice would afford us abundant illustrations; but the empirical artifices of the present day form still more familiar examples: to these I shall not particularly allude: some will *long* be notorious beacons.

From these sources of error, many useless, many nauseous, and not a few noxious, agents have, from time to time, been introduced, several of which have enjoyed an ill-earned fame; while some really-efficient medicines have as undeservedly fallen into disrepute. Hence, likewise, can we account for many of those superstitious rites, anciently so mixed up with medicine as to have been esteemed an essential part thereof. Few persons will take the trouble of distinguishing the *post* from the *propter*; and, even to those who would, the power is oft-times wanting. A mind, patient in observation, and well disciplined to distinguish truth from error, does not commonly coexist with that instinct (shall I call it almost blind instinct,) for generalization, by which theories are planned, and systems raised. Allow an example to illustrate this abstract proposition.

Achilles, writes the poet, escaped unhurt, though long exposed to all a warrior's danger, (and so did others of the Grecian force, and so do many others in every hostile meeting;) Achilles at last was slain by an arrow which transfixed his heel, (and so have many others fallen by wounds in some especial parts, whether in the head, the hand, the heel, for weapons to each victim are not omnipresent;) but Achilles had been bathed by Thetis, (and so by most parents have their sons been washed;) yet it is fabled that the heel by which his mother held him was the only part unwetted; that heel, it is said, was pierced; and hence arose the fame of the antivulneriferous waters of the Styx. "POST *ergo* PROPTER *balneum salus*."

Again, in times of general sickness, the Romans, with solemnity, elected a Dictator, for the especial purpose (and that alone) of driving a nail into the temple of Jupiter, and when afterwards the pestilence decreased, *post ergo propter malleum salus.*

Just as, at the present time, in countries where the plague prevails, an angel is believed to cast a drop of water on the earth, on the festival of St. John, after which day the plague is stayed, and to which the restoration of salubrity is attributed, rather than to the actual cause, viz. the great increase of heat that then ensues, and which is incompatible with its duration.

Again, honey was employed in ancient times, as still it is, as a useful application to relieve aphthous eruptions in the mouth and fauces; but then the relief obtained was attributed not immediately to the mean employed, but intermediately to an extraneous coincidence foreign to its nature, and only therewith fortuitously connected; i. e. the cure was ascribed by Soranus, who records a case in point, not to the honey, *as* honey, but to the accidental circumstance of *that* honey, which wrought the cure he mentions, having been procured from bees that had hived near Hippocrates' tomb.

Thus when men prescribed medicines, of the properties of which there was little known, for diseases, of the pathology of which they knew much less, it cannot be surprising that, although sometimes, *perchance,* they might assist recovery, more frequently they would do no good; and not uncommonly they would do much harm. Still, such was the perverseness of superstition, such the obtuseness of her votaries, that, whenever recovery ensued after the administration of any remedial means, were it either independent, or even in spite, of its effects, the cure was immediately attributed thereto; and when, as oftentimes occurred in cases of real disease, (although many slight or supposititious ailments would occasionally disappear during the exhibition,) it failed to cure or to relieve, some trifling variation in attendant circumstances, such as the mode or hour of administration or collection, or some such other trifling irregularity, not only foreign but impertinent to the question, was referred to as the source of failure: and hence arose many of those superstitious rites which figure so strangely in the medical records of antiquity. Thus, if, as we are told, the vervain cured the falling sickness in one individual, and in another failed; i. e. if *after* its administration one individual got better, and another worse, and if it was observed, or even fancied, that the latter had gathered the herb with his right hand, and the former with his left, this supposed ceremonial irregularity was esteemed a sufficient cause of failure; and hence arose the dogma that vervain should be gathered with the left hand only; but when, as could not but occur, of the left hand gatherers, some recovered and others died, further sources of disappointment were invented, and any thing was doubted rather than the potency of the plant *when charmed;* such as the time, the previous incantations, and other such like matters. But, in spite of all precautions, failures still ensued, and as fast as failures multiplied, so fast increased the ceremonials likewise, until at length we learn that the vervain was to be gathered with the left hand only, libations of honey being previously poured out, and after the plant had been encompassed with a circle: it was to be done likewise at the rising of the dog-star, and when neither the sun nor the moon shone. Again, the Selago was to be reverently approached with the feet bare, yet never to be touched with the naked hand. But, not to add instance to instance, for here the increase of examples is not required, the mistletoe, or all-health of the Druids, may be at once referred to, as a most remarkable and familiar illustration, and one, the particulars of which, without repetition, will be present to the memory of all.

When subsequently, as could not but occur, some refractory patients refused to take the nauseous doses which fraud and superstition had prescribed, and still, in spite of their presumption and irreverence, recovered, then the ceremonial observances of the friends or the attendants received the credit which had previously been given to the drug, and certain rites became esteemed as charms for the cure of persons sick, and for the preservation of those in health. Hence votive tablets and incantations sprang; in truth, a vast improvement in the art of physic; for, during these ritual observances, nature was allowed to effect a cure unimpeded by the interference of ignorant pretenders. The cure of wounds by sympathy forms of this a celebrated example; for thus the noxious dressings became applied to the weapon, instead of to the wound, which modest nature in the mean time healed; while incantations and sympathetic unguents gained the credit: to them, however, much praise was due, for the time they afforded to the *vis medicatrix* to effect a cure.

But were all the therapeutic agents, so famed in former times, utterly inefficient towards the recovery of health? Certainly not: far from it. But the principle of their administration was woefully misunderstood; and, consequently, the means themselves capriciously and often ineffectually applied. For who doubts the efficacy of hygeian springs, or the recovery of the sick who journey in quest of health to Hippocrates' tomb? Who doubts the history of strength restored to pious pilgrims, when abstinence and exercise (the sanative principles of the "*ball of basilisk*") were necessarily attendant on their visits to distant shrines. For one, I doubt not. I believe implicitly that many did not recover; that many were relieved; that many were restored to perfect health: just as I implicity believe a journey (call it, if you please, a pilgrimage,) to Cheltenham, to Harrowgate or Bath, to Madeira, the south of France or Italy, the change from a London to a country life, a necessity for exertion, more early hours, and often some slight abstinence in food, or a total change of diet, will even now, and in these degenerate days, do much towards checking the progress of disease, much towards the recovery of health. To be sure, the principles of the prescription are not similar, although both the agents and the patients are much the same. *Nomina* as well as *Tempora mutantur, sed nos* non *mutamur ab illis.*"

We smile at the recital of the superstitions and credulity of a former age, and we do well: though we little think that our smiles too often condemn ourselves: for how little do we yet know of the nature of the instruments we frequently employ for the cure of disease, and how very little of the nature of disease itself. I say not this invidiously, for much in both departments has been lately done; but this increase of light only the more strongly shows how very much more there remains for us to do. Still this is a subject rather of congratulation than despair; it is rather our incitement than disgrace.

Experimental study, supplanting mere speculative philosophy, has worked miracles on every side; and in no science has it wrought more wonders than in our own. Experiment is the key by which we have been already able to translate so many passages from the book of nature into our mother tongue, and by which we hope to be enabled to construe many more. But because much has been lately done, therewith shall we rest content?—forbid the unworthy thought; for contentment too often is allied to sloth, and sloth is the bane of science. Our predecessors have been our pioneers; they have cleared away from the entrance to the temple of Truth much of the accumulated rust of ages. We now no longer hear of the royal touch being efficacious in the cure of scrofula; although once, in this very town, a philosopher (who lived, as we might say, before his age,) was tried, found guilty, and severely punished, for doubting the efficacy of this repellent. We no longer hear of those disgusting tales of females crowding to the gallows' foot, to have unsightly tumors rubbed with an executed felon's hand; and their necks, by the officiating hangman, smeared with perspiration wrung from a fellow-creature in the agonies of death. No longer are our Dispensatories disgraced by the enumeration of "manus regalis" et "manus hominis mortui" as remedial means. Yes: our Dispensatories have been much purified of late, and medicines no longer are prescribed merely because they are nauseous; nor things now given to a man when sick which would disorder his system even in the most perfect health. General observations are, however, tedious: let them be changed for an example. Among the much-valued vegetable medicines of the century before the last, was mouldiness, or mildew: not simply mould, not that which springs on decaying wood, or bread, or meat, or cheese, but mouldiness scraped from a human skull. This drug even stands in the catalogue of materia medica published by the London Royal College of Physicians A. D. 1618, as "usnea cranii humani," which shows how much it was esteemed; for the College have ever striven, as far as the prejudices of the age would let, to discard all useless articles, and to march, though with discretion, not hardihood, in the van of science. Again, the urine and the excrement of various animals were formerly much esteemed: and of each we find extensive lists recommended officially to notice. Much of the supposed efficacy of drugs seems once to have been attributed to the filthiness of the sources whence they were derived: thus, although carbonate and phosphate of lime are excellent absorbent earths, and exhibited with much benefit in many cases of cardialgia, diarrhœa, and other complaints, still a simple chalk mixture would have been formerly despised; while one of *album græcum* would have been swallowed with avidity. Now album græcum, it is all but needless to observe, was only an impure bone earth, not obtained from the laboratory of the chemist, but elaborated in the intestines of the dog. For the preparation of this precious drug, dogs were purposely all but starved, well-picked bones being their only food; and then, the gelatine of the bones being partly absorbed during the passage of this osseous matter through the alimentary canal, the earthy portions were evacuated in a half-blanched and semi-pulverulent condition: this excrement was then collected with a jealous care, and became the album græcum of the shops, highly prized as a discutient, and forming a favorite internal application to the throat in quinzies. I will not waste your valuable time in further disquisitions on this subject, although it is a point of curious interest to trace the progress of the art we study, and highly useful sometimes to unravel the absurd intricacies of those who loved to make medicine a greater mystery than it ever will inevitably be; for thus do we in our minds enhance the estimation of that unsophisticated and simple nomenclature which signalizes the age, and with which science now in general is blessed: and we shall ever value more this striving after perspicuity, even if it fail, when we contemplate the obscurity in which all subjects were at one time purposely involved; *names* being given to *things* apparently rather to conceal than to explain their nature, although sometimes, as in the *aqua omnium florum*, there was an oracular latent witticism which fails not to provoke a smile; for, says BAILEY, "*aqua omnium florum*" means (among chemists) the distilled water of cows' dung, when cows go to grass. But of this enough: one sample of such precious balms may well suffice.

An imperfect knowledge of the essential properties of medicines, especially of the vegetable materia medica, has always led, as still it does, and as long as it exists must ever lead, to complicate our prescriptions, to cripple our resources, and to confuse our principles of practice. Formerly this was much more prevalent than it can be now; but, even in the present age, I doubt not that great and unneeded complexity remains, though as yet it may be hidden from our eyes. Each succeeding year long has tended to simplify and curtail our catalogues of drugs; and each succeeding year, it may be easily foretold, will tend to simplify and curtail them more. I could point out many instances, both ancient and modern, in which agents, similar in effect, if not identical in composition, appear many times under different denominations, their names being the points in which they differ most. How many more varieties of carbonate and phosphate of lime do we even now admit, merely because they are derived from different sources, and how much more numerous were they in the age before the last, when all drugs were considered essentially distinct whenever the substances were distinct from which they were obtained. I have chosen this purely chemical illustration because chemistry has lately thrown so much light on medicine, and promises to shed much more, and its researches are far more complete in the constitution of inorganic nature. Indeed, we are at present unable to determine, in the vegetable department, how far many of our most costly drugs may be entirely dispensed with. How many more varieties of expensive astringents, bitters, aromatics, &c. do we not now employ, when more than a reasonable doubt may be entertained as to whether, as in the case referred to, their truly efficient principles may not be extracted from cheaper materials and more abundant sources: of this we have already proved the practicability in several important cases. Thus, the vegetable alkali, our sub-carbonate of potash, was at one time possessed of numerous names, and appeared and reappeared over and over again in the Dispensatory; and yet, at each repetition, seems to have been considered a new and independent drug. Thus, we find it introduced under the style and titles of Sal Absinthii, or salt of wormwood, Centaurii minoris, Cichorii, Euphrasiæ, Fabarum, Fumariæ, Genistæ, Herbarum, Plantaginis, Plantarum, Querens, Tartari, and many others, as each vegetable was considered to afford a peculiar salt: while, in truth, none of them differ essentially from each other, the only variation being in their relative degrees of impurity; so that the terms Sal vegetabiles, or Sal plantarum vel herbarum, would conveniently include the whole. And yet, even now, or very lately, as I have been credibly informed, some persons will give a quadruple or quintuple price for Sal absinthii, an impure subcarbonate of potash, obtained from wormwood, to what they will for the same, but more pure and valuable drug, derived from common and less costly sources.

This is but a solitary example; yet " ex uno disce omnes."

Shall I add a further illustration, *oxalic acid*, if procured alone from the plants whence sprang its name, (Oxalis autosella, Corniculata, &c.) would be a very expensive drug: even when at first, under the cognomen *Acidum sacchari*, it was formed by the destruction of sugar, it was, within my recollection, sold in this town at 3s. 6d. and 4s. per ounce; but now it may be bought at the manufacturing chemists for about half that sum per pound.

Probably a like fate awaits many of the proximate principles, in modern times discovered, and still considered to be distinct. Thus, colchicum is doubtless a useful medicine, though fashion has somewhat enhanced its fame, while Gratiola, the old Gratia Dei, and Veratrum album, or white hellebore, have completely fallen into disuse, if not into disrepute; and yet the active principle is in all the same, and might, if extracted from either, be indifferently used, and with equal advantage and effect. May not this indicate the propriety of extracting veratria from hedge-hyssop, veratrum, where those plants abound, rather than from the expensive colchicum? These instances must likewise indicate the advantages likely to accrue from an intimate acquaintance with the proximate principles of vegetables, when we may find our active agents, not in one situation only, but recognize them in many, and seek them wherever they may be most abundantly present, or most easily be found.

I fear that an accumulation of the evidence of how much is wanted to be known on the points which this Society has proposed as the objects of its chief inquiry, might weary the attention of the meeting. I will, therefore, add but one example more,—the value of Peruvian bark; it is likewise known to every one that there are many species of cinchona, differing in their efficacy and worth. Of these, three are admitted into our London Pharmacopœia; viz. Cinchona lancifolia, or lance-leaved cinchona, affording the pale brown bark; C. oblongifolia, or oblong-leaved cinchona, yielding the red bark; and C. cordifolia, or heart-leaved cinchona, which gives the yellow bark. Now, of these three, it is well known that the lancifolia, or quilled bark, has in general been, by the profession, the most esteemed; still the oblongifolia, or red bark, has not been without its advocates; indeed, popularly, it was so highly prized that, in the markets, it would occasionally fetch threefold the price of brown, and six times as much as the yellow, or heart-leaved species, which was comparatively slighted both by the profession and the public. Perhaps, the scarcity of the red bark contributed something to its high estimation, as the relative abundance of the yellow not improbably increased its disfavor, just as at the present day no one will use, (indeed, who would condescend to use,) the indigenous simple, aromatic, and astringent bitters, Nenianthes, Teucrium, Salix, Acorus, and Bistort, which grow abundantly at Hampstead, Highgate, Battersea, and Paddington, when they can have Quassia, Catechu, Cinchona, and Colomba, by sending to Hindostan, Jamaica, Mozambique, or Peru?

And here (as in a parenthesis) I would observe, that our native medicinal herbs have of late been too much neglected; for certain it is that we compass half the globe to import a drug, the prototype of which not unfrequently solicits our hands at home. I, for one, can never think that all those are useless that we use not; that such countless myriads of beauteous herbs which spring profusely wild over all the deep green earth, spring oft in vain, because in vain they court man's notice and regard; I never can believe that Providence has armed the weeds of foreign lands with powers necessary for us, whilst ours are impotent to heal. I never can believe our herbs inert, whilst every plant in other climes may boast itself a physician's staff. I know that it is by a merciful dispensation that man has been condemned in the sweat of his brow to eat his bread; I feel that, where necessity compels not to exertions, indolence debases man almost to the level of a brute; and likewise, I confess that, where most is required for the body, there most fully are developed the energies of the mind; still I cannot but perceive that many of our native plants want but to grow upon the Andes or the Alps to be sought with avidity, and treated with respect.

It is therefore a matter of no mean importance to which, by a resolution of the council, the attention of the scientific world at large, of the members of this Society in general, and of my colleagues and myself in particular, is directed, by the determination to award the silver medal of the present year to the author of the best essay on the nature and properties of any indigenous plants the medicinal powers and properties of which have been hitherto unknown, or only imperfectly recorded. To me it appears that this will be sinking a shaft in a very rich mine of scientific discovery, of truly philosophical research. I doubt not, that we shall find many of the remedies now sought from the tropics and the poles growing at our doors, and soliciting to be allowed to relieve our ills. It is an amiable idea, and one that experience goes far to verify, that wherever natural circumstances favor the production of disease, Nature, i. e. nature's God, hath beneficently conjoined the means of cure. May not the late experiments with Salicine be given as an apposite illustration? For, although we have so long been ploughing the Atlantic, and burdening the bosom of the deep, to bring home our harvests of Peruvian ague-cure, the ever valuable cinchona, *Salicine,* extracted from our native willows, so far as experiments as yet have gone, is proved to be equally efficient with the *Quinine* of Peruvian bark. Where do agues most commonly prevail? Where do we find remittent and intermittent fevers of the greatest frequency and most fatal severity? Where! but in wet, low lands; in marshy and in fenny districts. And where do willows love to dwell? Where! but in those very fens and marshes; as if to relieve the diseases inseparable therefrom. Hence I cannot but regard them as the living elaboratories of nature, thus planted by Providence to form Salicine for the use of man.

(To be continued.) p. 287.

